# Adjustment and coping in spousal caregivers of cervical cancer patients in Ghana: A qualitative phenomenological study

**DOI:** 10.1097/MD.0000000000038807

**Published:** 2024-07-05

**Authors:** Evans Appiah Osei, Isabella Garti, Mary Ani-Amponsah, Elvis Frimpong, Hawa Amadu Toure, Jamilatu B. Kappiah, Manuela Akosua Menka, Samuel Kontoh

**Affiliations:** a School of Nursing, Purdue University, West Lafayette, IN; b University of Charles Darwin, Casuarina, NT, Australia; c Maternal & Child Health Department, School of Nursing and Midwifery, University of Ghana, Legon, Ghana; d Shai-Osudoku District Hospital, Accra, Ghana; e Ghana Health Service, Accra, Ghana; f School of Nursing and Midwifery, C. K. Tedam University of Technology and Applied Sciences, Navrongo, Ghana; g Valley View University, Accra, Ghana; h School of Nursing and Midwifery, Valley View University, Oyibi, Ghana.

**Keywords:** cervical cancer, diagnosed, Korle-Bu Teaching Hospital, males, partners

## Abstract

Cervical cancer is a common and significant health issue for women worldwide. To address the dearth of research on male partners’ experiences when their significant others are diagnosed with cervical cancer, we aim to explore the unique challenges and perspectives encountered by men in these circumstances. The study adopted interpretive phenomenological analysis to qualitatively assess the experiences of males with partners diagnosed of cervical cancer. A phenomenological research design with purposive sampling technique was used to recruit and collect data from 38 participants until saturation occurred. Face to face interviews were conducted using a developed semi-structured interview guide. The data collected was analyzed using content analysis after verbatim transcription was done. The study resulted in the identification of 2 main themes, and 10 subthemes. These themes focused on the multifaceted impact of cervical cancer on spousal caregivers’ lives and the coping and support mechanisms utilized by spouses of cervical cancer patients. The findings indicated that men faced several challenging experiences as a result of their spouses’ condition and revealed the strategies they employed to cope with the stress of caring for their wives. Almost every man adopted a strategy to cope with the condition of their wives. This study would assist other men to understand the psychological, social, emotional, and spiritual experiences the men went through to appreciate and adopt their coping strategies whenever they go through such challenges.

## 1. Introduction

Globally, cervical cancer has been rated as the fourth most common cancers affecting women.^[1,2]^ In the United States, it has been estimated that about 12,200 new cases of cervical cancer were diagnosed in 2010, with an incidence rate of 7.9 per 100,000 women.^[3]^ The American Cancer Society has predicted that about 14,480 new cases of invasive cervical cancers will be diagnosed and about 4290 women will die from cervical cancer in the next 5 years.^[4]^ Cervical cancer is a major cause of cancer mortality among women, and more than a quarter of its global burden is contributed by developing countries.^[5]^ According to the World Health Organization, it is estimated that there are over 500,000 new cases of cervical cancer worldwide.^[6]^ Being the partner of an individual diagnosed with cervical cancer entails encountering novel challenges and assuming additional responsibilities concerning emotional fluctuations and rehabilitative efforts.^[7]^ It is noteworthy that literature pertaining to the experiences of males with partners afflicted by cervical cancer worldwide remains scarce. Upon a woman’s diagnosis with cervical cancer, the couple frequently experiences a sense of helplessness.^[8]^ Partners of women diagnosed with cervical cancer contend with various challenges as they endeavor to provide emotional and financial support to navigate the adversities associated with the condition.^[9]^ Furthermore, many men contend with feelings of depression, anxiety, or loneliness while caring for their partners grappling with cervical cancer.

Encountering physical, psychological, economic, and social obstacles in their daily lives as male partners, have a toll on their quality of life as well as that of their partners.^[10]^ Understanding the experiences partners of patients diagnosed with cervical cancer will help determine effective interventions that can improve cervical cancer care.^[11]^ According to,^[12]^ chronic diseases such as Human Immunodeficiency Virus/Acquired Immunodeficiency Syndrome and cervical cancer can change the life events and biography of patients and their partners. In other words, chronic disease like cervical cancer can interrupt the lives of patients, hence, the patients need extra care and full support from partners and other family members to help them cope with pain and suffering associated with the condition.^[13]^

In Ghana, it is estimated that 3052 new cervical cancer cases were registered in 2012, accounting for 32.7% of the country total female cancer cases.^[14]^ Tracing the path of male partners of cervical cancer patients unveils a progression marked by phases of physical, psychological, economic, and social upheaval—a collective response ignited by the culmination of the disease’s impact.^[15]^ Furthermore, the patients’ capacity to participate in their customary daily activities becomes a subject of concern. Both the patients and their partners grapple with psychological upheaval, as they navigate an integral scenario steeped in profound uncertainty.^[16]^

### 1.1. Research questions

1. What are the multifaceted impacts of cervical cancer on spousal caregivers’ lives of partners diagnosed of cervical cancer at Korle-Bu Teaching Hospital (KBTH)?
2. What are the coping and support mechanisms for spouses of cervical cancer patients?

## 2. Methods

### 2.1. Design and setting

This study used a qualitative method because the research aimed to explore the experiences of males with partners diagnosed with cervical cancer at KBTH in order to gain a profound and in-depth understanding of their experiences. Phenomenological research design is described as a type of qualitative research design where lived experiences of persons within the world are studied.^[17]^ This study employed a phenomenological research design to enable participants/males to come out with their real-life experiences about their women living with cervical cancer. The study was conducted at KBTH. KBTH is a public teaching hospital located in the Ablekuma South Metropolitan District in Accra, Ghana. Korle-Bu which means the valley of Korle lagoon, is currently the third largest hospital in Africa and the leading national referral center in Ghana.

### 2.2. Study participants

This study targeted men whose partners were undergoing treatment for cervical cancer at Korle-Bu Teaching Hospital and who were proficient in English, Twi, or Ga for effective communication. Excluded were males with severe illness or psychological trauma, and those who were deaf or mute but had partners with cervical cancer. Purposive sampling, or “judgment sampling,” was employed to deliberately select participants who met the study criteria. Only men whose partners had been diagnosed with cervical cancer were eligible. The sample size, crucial for making population inferences,^[18]^ comprised 38 participants, as data saturation occurred before reaching the 40th participant, despite 42 being contacted, with 2 declining participations.

### 2.3. Data collection instrument and procedure

A semi-structured interview guide served as the primary data collection tool, tailored to the research objectives and relevant literature issues. A pilot study, involving 3 consenting participants with partners diagnosed with cervical cancer at Shai-Osuodoku District Hospital, was conducted to refine the guide based on feedback. The experiences of these 3 participants were not incorporated into the final analysis.

At the designated venue and scheduled time, the researcher convened with participants who fulfilled the specified inclusion criteria, guided with a semi-structured interview guide. Engaging the participants, the researchers introduced themselves and delineated the study’s objectives in English. Given that all participants were proficient in Twi and Ga, in addition to English, the decision was made to conduct all interviews in English.

Informed consent was obtained prior to conducting face-to-face interviews with each eligible participant. The researchers ensured a conducive environment, free from disruptions, where participants were comfortably seated. Beginning with rapport-building, discussions centered around the study topic: “Experiences of males with partners diagnosed with cervical cancer.” Interviews, lasting approximately an hour, were recorded for accuracy. Data collection continued until saturation was reached, indicating no new information emerged. Participants were thanked at the conclusion of each interview, with follow-up calls made as necessary for clarification.

### 2.4. Ethical consideration

Ethical clearance was obtained from Dodowa Health Research Centre Institutional Review Board with the protocol number DHRCIRB/037/03/22 before data were collected. The purpose of the study was explained to the participants. Consent was obtained from participants before engaging them to participate in the study. Participants were informed to freely withdraw from the study without any penalty as and when they deem fit. All other ethical principles regarding human research were maintained and adhered to.

### 2.5. Data analysis

Phenomenological analysis (PA) is a qualitative approach which aims to provide detailed examinations of personal lived experience.^[19]^ This study used IPA in accordance with the topic statement: “experiences of males with partners diagnosed of cervical cancer” in order to explore their lived experiences to make the topic a success. The researcher’s own interpretation was generally accompanied by a deep study of personal experiences, followed by presenting and debating the generic experiential themes, which was an example of double hermeneutics in practice. Researchers obtained qualitative data from study participants utilizing procedures such as interviews, diaries, and focus groups in PA data collecting. Typically, they are approached from a position of open-ended and flexible inquiry, with the interviewer using a curious and facilitative attitude.

Personal accounts of some richness and depth are normally required by PA, and these accounts were documented in a fashion that allowed the researcher to work with detailed verbatim transcript data annotating it meticulously (coding) for insights into the participants’ experience and viewpoint on the matter, which were recorded and saved on a personal pen drive. The researcher catalogues or lists the new codes as the investigation progresses, and then begins to seek for patterns in the codes. These patterns are referred to as “themes. Themes in a text are repeating patterns of meaning (ideas, thoughts, and feelings). The themes are; phenomenology, hermeneutics, and ideography. The recorded interviews were transcribed manually and the soft copies of the transcribed responses were saved on the researchers” personal laptop with a password and the signed consent form kept at a safe place and locked by the researcher. Every data obtained was treated confidential. Content analysis was used to analyze the data.

### 2.6. Methodological rigour

The term “methodological rigour” refers to a set of factors that, when taken together, determine the level of confidence with which conclusions can be formed from evaluation results.^[20]^ In qualitative research, rigor means making the research strategy, technique, and results plain, public and reproducible, open to criticism, and bias-free. Lincoln and Guba established strict qualitative research standards known as credibility, dependability, conformability, and transferability in order to establish trustworthiness. This is referred to as “the Four-Dimensions Criteria” in this study. Credibility was accomplished by verifying that participant met the inclusion and exclusion criteria. Participants were given a thorough explanation of the informed consent process, as well as answers to any questions they had. To ensure dependability, the study’s environment, phases, and methods were thoroughly discussed. This included a review of the study’s findings, interpretations, and recommendations to ensure that the data collected from participants was reliable. For conformability, the researcher considered his own beliefs, personal feelings, biases and insight to ensure that they did not influence the study’s findings throughout the interview. In the field diary, the sample size and data collection process were noted. Direct quotes were utilized to support emergent themes after audio recordings were transcribed verbatim, as well as engaging in an audit trail. While transferability was also maintained by providing a full description of the technique and processes used to conduct the interviews.

## 3. Results

### 3.1. Socio-demographic characteristics of participants

The total number of respondents in this study was 38 with the age range from 38 to 72 years. Less than half (31.6%) of the participants were below age 51 and the rest (68.4%) of the participants were above age 50. The religious affiliation of the study participants indicated that 7 (18.4%) of participants were Muslims, 29 (76.3%) participants were Christian and the remaining 2 (5.3%) are other religions. This is not surprising, because in Ghana, the Christianity is the dominant religion. Refer to Table 1 for detailed demographic information on the participants.

**Table 1:** Socio-demographic data characteristics of respondents.

Variable	Frequency (n = 38)	Percentage (%)
**Age group**		
38–40	3	7.9
41–50	9	23.7
51–60	16	42.1
61–72	10	26.3
**Religion**		
Christian	29	76.3
Muslim	7	18.4
Other religion	2	5.3
**Marital status**		
Cohabitation	4	10.5
Married	34	89.5
**Number of children**		
0	3	7.9
1	7	18.4
2	9	23.7
3	5	13.2
4	8	21
5	6	15.9
**Educational level**		
JHS	8	21
SHS	13	34.2
Tertiary	11	29
None	6	15.9
**Occupation**		
Farmers	14	36.8
Accountant	3	7.9
Business	13	34.2
Mechanics	5	13.2
Health-worker	1	2.6
Teacher	2	5.3
**Ethnicity**		
Ga-Adangbe	6	15.9
Guan	1	2.6
Akan	18	47.3
Ewe	11	29
Gurma	0	0
Mole-Dagbani	2	5.2

### 3.2. Organization of the themes

From the data collected, there are 2 major themes. The themes were grouped into 10 subthemes. The major and subthemes are presented in Table 2.

**Table 2:** Themes and emerging subthemes.

Themes	Subthemes
1. The multifaceted impact of cervical cancer on spousal caregivers’ lives	1. Intimacy challenges in relationships 2. Adaptation and adjustment to caregiving responsibilities 3. Mental health struggles 4. Spiritual and existential questioning ** 5.** Loss of interest in once-enjoyed pursuits ** 6.** Concerns about personal health and risk of cervical cancer transmission
2. Coping and support mechanisms for spouses of cervical cancer patients	7. Adaptive coping strategies and multifaceted support systems receiving assistance from healthcare professionals. 9. Receiving support from social networks 10. Seeking solace in spirituality/religious beliefs

### 3.3. The multifaceted impact of cervical cancer on spousal caregivers’ lives

#### 3.3.1. Intimacy challenges in relationship

As a matter of fact, sex play a major role in relationship especially in marriage, it serves the purposes as source of happiness, reproduction, entertainment, exercise, boosting morale and confidence. Most of the males were of the concern that their wives could not satisfy them sexually due to the pain they go through during sex.

*“I find it difficult of late to have sex with my partner, initially when the disease was not there we could have sexual intercourse for about two or three times in a day at least one with each not less than one round which she was really enjoying it. But now not even once, she will complain bitterly when we have sex”.* **P19**

*“My wife is denying me sex because of the pain she goes through during and after sex. If I remember this, it takes my libido away. This has become my burden, I can’t also cheat on her, it is making life difficult at home. Hmmm, life without sex is full of temptations that one need more grace and strength to pass through. It is not easy my brother”.* **P10**

*“In fact when it comes to sex life I’m really lacking, no enjoyment, if I try and persuade or convince her to have sex, she goes through pain, it makes me feel guilty and regret after sex so I have stop having sex with her for 4 months now”.* **P4**

Some males were psychologically unstable because of the condition and state their wives were going through which never made them think of sex let alone to do it.

*“I am not even thinking about sex, my wife is dying and I spent all my money on her and she is still not well. I am not psychologically stable how can I think about sex”* **P28**

### 3.4. Adapting and adjustment to caregiving responsibilities

When it comes to the home, most of the work (house chores) such as caring for the kids, washing, cooking, general cleaning is taken care of by the women in our African setting and sometimes supported by the man. Hence sickness of the woman led to extra workload on the man. This issue was reported by the men that they were overburdened with household duties and care of children because of their wife’s sickness.

*“Is not easy for me in the house, I have asked my wife not to do anything because of her condition and I am the one doing everything. It hasn’t been easy for almost 8 months now, but she has been a good wife, I have to support her”.* **P 29**

*“Well, my children have now become a burden on me now, you know, my last born is just about 3 years old. Initially, whenever she is going to school. The mother baths and dress her, but now that she is sick, I have to do all those things. The house chores are also on me. I get exhausted combing all these with work”.* **P3**

*“At times, I feel like she shouldn’t do anything which is Um, let me say a bit tedious to do. So, I have to always make sure I’m around to assist her and taking care of the home. And at times, I have to do some of the house chores so that it will release her from stress and fatigue. Any small work then she is tired and weak unless she rests for more minutes*”. **P10**

### 3.5. Mental health struggles

Major life event happens to be one of the main causes of depression aside genetics, substance abuse, and early childhood experiences. Disease is an environmental stressor and a source of distress that affects the individual and the relatives biologically, psychologically, and physically. Among those causes, being diagnosed of a chronic disease is one of the main causes of depression since it affects all aspect of an individual’s life. It was therefore not surprising that some of the men established that they were depressed.

*“Since my wife had this condition, I have not been able to cope as a man, I have kept worrying, sometimes I have to lock myself up and cry because I am a man and I don’t want people to know”* **P3**

*“I have always found ways to cope with any condition of life but this thing is too much for me. How can my life be affected with this condition? I feel helpless, I don’t know what to do”.* ***P29***

The situation of depression made other men who are Christians sought the face of God regularly through prayers for healing for their wives to have tranquility in the family as it was initially before the condition came.

*“Am not stable emotionally, always thinking hard. Feeling bad and weak sometimes. Things are hard. I pray she gets well soon so that things fell in their rightful place again. I often see the pains my wife goes through and is not easy for me even as a man. I am a Christian and praying and hoping for healing one day. As a family, we are worried a lot at times. So, she cries now and then we are broken hearted”*. **P12**

*“Once in a while I feel so exhausted and down since I know this is a serious condition. Furthermore, things have become very hard. So, it has not been simple as far as we’re concerned sincerely by any means. I mostly wake up in the midnights and I can’t sleep again, thinking in series. Whenever I’m awake, I consider it and think through how possible this happened to my wife how did it start to come about and makes me ask God when! So I generally give everything to God”.* **P7**

### 3.6. Spiritual and existential questioning

Some life challenges cause others to lose their faith whilst it increases the faith of others in their Supreme beings. Since majority of the participants were Christians, they reported how their faith in God strengthened following the diagnosis.

*“As I am a Christian and the Bible makes us understand that in all things by prayer and supplication make your intentions be known unto God. This statement empowered me to exercise my faith in God by praying consistently and persistently asking God Almighty to intervene to heal my wife. Even though she is not healed yet but I have not lost hope and faith in God”.* **P34**

Few Christians were also disappointed in God for their unmet expectations.

*“I have prayed and believed in God for my wife’s healing for years now but situation remain unchanged and even getting worse. What I know as a Christian is that, all power belong to God and he can heal every disease including cancer by the blood of Jesus Christ if I believe in him. I have believed and prayed consistently but my wife was not healed so I have loss hope and stopped praying since nothing comes out from it. I’m highly disappointed in the failure of God’s healing power”*. **P25**

Participants who were Muslims also expressed their disappointment and how the disease affected their faith in Allah.

*“Any day I do my prayers, I remember my wife and I have asked my other wives to also remember her in their prayers. Upon all these prayers we say for her, her condition does not improve, for more than 2 years we have been praying as she suffered from the cancer. I’m even fed up and lose interest to pray for her again because I know Allah has heard it but he chooses what to do and doubt his love for my family. For this reason I have left everything in his hand if she would be healed fine if not it is up to Allah”.* **P35**

Finally, a participant who neither believe in God or Allah also expressed what he believed about the disease.

*“I know that everything that happens in life is bound to happen and it is by nature, you have no control over it. If my wife get healed it is one of those things and if she dies that is her fate. After all, we will all die one day”.* ***P7***

### 3.7. Loss of interest in once-enjoyed pursuits

Every person has activities/recreation enjoyed. Most individuals facing life challenges resort to these activities enjoyed by that individual to cope with that particular situation. Nevertheless, the men in this study recorded loss of interest in previously enjoyed activities.

*“I remember the early days of our marriage even until when the sickness came we were really enjoying our sex life together as couple, because of her situation now, even if we are able to have sex I do not get any enjoyment due to the complaints and the trauma from the disease. I don’t fancy sex anymore”.* **P16**

*“I’m the outing type, I like moving out with friends to enjoy ourselves but since this condition, all that feelings of going out to places like beach, pubs, stadium, parties, and the likes have all gone. I cannot even concentrate on that”.* **P23**

*“I use to enjoy spending time with relatives and friends to chat but because of the condition, I have to spend all the time with my wife and I don’t feel like chatting with friends”.* **P20**

### 3.8. Concerns about personal health and risk of cervical cancer transmission

There are 2 main classifications of disease which are communicable and noncommunicable disease. Some diseases are infectious and that they easily transfer from one person to the other such as chicken pox which comes under the communicable diseases. Some of the participants had the notion that cervical cancer is an infectious disease like any other sexually transmitted diseases like gonorrhea, syphilis, chlamydia, Human Immunodeficiency Virus/Acquired Immunodeficiency Syndrome, and among others but cancer is not infectious disease. The men expressed their fear to contract the cancer if they have sex with their partners.

*“I can’t have sex with her, no, no. One, she would be in pains and again if someone is diagnosed of cervical cancer the likelihood that she had the human papillomavirus is high. So I don’t want to also contract any cancer. Prevention is better than cure as they usually say. I will let her have her peace, my brother”.* ***P21***

*“My wife has been having diseases and odor smell because of her condition so I think if I have sex with her I will also get the cancer. This made me stopped having sex with her unless may be I use condom”.* ***P8***

*“I have had an STD before during my 20s and how I suffered. I was the shy type so I kept the sickness to myself buying pain killer to control pain until I was no longer to keep it which was getting worse. I later confided in a pharmacist until I got solution. Just one sex and one night took me that far of pain. When I was told the diagnosis was cervical cancer how I was scared having sex with her might predispose me to the condition”.* **P36**

### 3.9. Coping and support mechanisms for spouses of cervical cancer patients

#### 3.9.1. Adaptive coping strategies and multifaceted support system

Sometimes it is very needful to use no drug to treat patient illness because some illness do not require drugs to treat and drugs have numerous side effects. At times, a person just needs counseling, motivational speech, nutrition, exercise, sleep, water, and diversional therapy to help manage his/her condition. This diversional therapy takes the mind of the patient out of the sickness and help to relieve the patient from the pain she is going through.

*“Yes, one thing she like best is telenovela. There is one particular program that the whites speak Twi language. It is her favorite program on TV so I have bought some of the CDs for her to watch to help her relieve her of anxiety and overthinking. Again, when they are showing some on TV, I call her that her program is about starting then she quickly come for us to watch together in happiness.”* ***P29***

*In fact, she likes games. So, any time she’s in pain upon taking her due medication, I try to bring back the game that she likes. Before we got married or before the condition, she loved a particular video game that are on her phone, so any time she exhibits those signs and symptoms, as part of the treatment, I quickly tune in her mind back to the game, we will play, then she will fall asleep*”. **P27**

*“I bought ludu and oware to use as indoor games in our home for the sake of my wife to keep her company. When it happens that she is very quiet I have noticed that she start to think and reflect on the effect and the impact of the cancer which is not a good scene to me. Therefore, I play any of the games of her interest or choice to take away her mine to enable me cope well, otherwise that can make me sad throughout the day”.* **P33**

### 3.10. Receiving assistance from healthcare professionals

Support comes in various forms such as money, advice, emotional help, and the likes from various personalities. Participants in this study revealed that health care providers form active part in provision of support to women with cervical cancer and family.

*“First of all, I want thank all the health workers in this department, in fact they have done well for me, most of them supported me emotionally, some even went as far as long to support me with some money, just last one of the nurses gave 150 Ghana Cedis to buy diapers for my wife, they are very supportive”* ***P6***

*“The health workers are very supportive, they always give us hope, they offer counselling to us, they are very good. Kudos to them”* ***P8***

***“****The health workers here are amazing, they have being helping us a lot, they talk to us with dignity. They are very good people”.* ***P13***

### 3.11. Receiving support from social network

Difficult and challenging situation helps one to know his or her time friends. Time friends usually support their friends throughout challenging times whilst fake friends do otherwise. Another important finding from this was support from friends and relatives. Some of the participants said they have received financial support from their relatives and friends. Some of their expressions were as follows;

*“I cannot thank my family enough, they have been supporting me since the onset of my wife’s condition, they have contributed more than 5,000 Ghana Cedis (627-USD) for me to support my wife. I can’t than them enough, may God richly bless them”* ***P9***

*“Brother! Is good to have friends, my friends have helped me a lot, I have received more than 10,000 Ghana Cedis (1,255-USD) from my friends. Some of them also have encouraged me a lot, giving me all forms of reassurances to console me”* ***P7***

Few of the participants also said they have not received any support from family or friends.

*“Truth must be told, I have not received any financial support either from my family or my friends, I do everything on my own, and I don’t have problem with any of them, so if they bring it, I will gladly take it and thank them.”* ***P12***

### 3.12. Seeking solace in spirituality/religious beliefs

There is a belief that prayer answers almost all things; being faced with challenges draws people close to the creator. Most people lose interest in material things and become more spiritual. It was therefore not out of order that participants, especially the Christians narrated that they draw closer to God throughout their situation.

“*We pray a lot since the sickness started because I learned the cancer cannot be cured so our only hope is in our Lord to heal her. This has even made us more prayerful than before. We also ask our pastors, prayer group and friends remember us in prayer because we believe god is a mighty healer*.” **P1**

“*Oh yeah, because in everything, God, first. Doctors are doing their best, but I think, the great healer is God himself. He has given wisdom to doctors to diagnosed and prescribed medications, so the source of everything is God. So I still rely on him that one day or very soon he will heal my wife. I pray to God every day to intervene in her situation, Yeah*.” **P27**

“*We frequently seek the face of the Lord in prayers. Since we learnt that cancer cannot be cured so our only hope is in our Lord to heal her. This has even made us more prayerful than before. We frequently seek the face of the Lord in prayers. Since we learnt that cancer cannot be cured so our only hope is in our Lord to heal her. This has even made us more prayerful than before.”* **P17**

In the Muslim fraternity some men showed their faith through consistent prayers and hope in Allah.

“*Okay, I believe everything is by Allah. …..Maybe she is destined to have that sickness all is in the plan of Allah, if he does not allow it, it will not happen. Let his will be done. That doesn’t affect my faith in anyway. I still pray and believe in Allah uninterrupted by this current situation. Allah is the supreme healer of any sickness so I have left everything in the hands of him to take care. My hopes and faith in Allah are keeping me with her*”. **P10**

## 4. Discussion

One notable finding from the study is that many men reported encountering intimacy challenges stemming from their wives’ complaints of painful sexual intercourse, resulting in the cessation of sexual activity within their marriages. Given the fundamental role of sex in marital relationships, its absence can contribute to marital dysfunction. Furthermore, men who struggle to manage their strong sexual urges may benefit from additional counseling to effectively support their wives psychologically. These findings resonate with research conducted in Norway,^[21]^ which similarly highlighted significant alterations in relationships, particularly concerning sexuality, often due to the experience of painful intercourse and subsequent disinterest in sex among women.

Many participants expressed the need to adapt and adjust to cope with the increased household workload resulting from their wives’ ill health. This adjustment includes assuming responsibility for various household tasks such as childcare, cooking, and general caregiving alongside their own work commitments to finance their wives’ treatment. For those with the means, employing a reliable house help is recommended, while families are encouraged to provide maximum support to help men navigate this challenge effectively. This finding aligns with prior research,^[22]^ which found that women in relationships characterized by perceived unequal distribution of household responsibilities experience higher levels of stress, fatigue, physical/psychosomatic symptoms, and work-family conflict compared to those in more equitable relationships.

One significant finding of this study pertains to the mental health challenges experienced by male partners of women with cervical cancer. Many participants reported feelings of depression stemming from their spouses’ illness, describing profound sadness and emotional distress. Consequently, men in such situations require comprehensive support mechanisms including effective counseling, positive reassurance, motivational guidance, and assistance from their social networks. This observation aligns with prior research,^[23]^ which identified depression as a prevalent psychological issue among women with cervical cancer. The similarity underscores the need for holistic support interventions to help both partners navigate the emotional toll of the illness effectively.

The diagnosis of cervical cancer had a positive impact on the spirituality of many participants, leading to an enhancement of their faith. Several Christians reported a deepening of their prayer life and a closer connection to God, intensifying their hope for divine intervention in their wives’ healing. This spiritual fortification served as a coping mechanism, helping them navigate their anxieties. This finding resonates with a study in Hong Kong^[24]^ which explored the significance of spirituality among Chinese individuals, emphasizing its role in fostering inner well-being and resilience. Conversely, some men expressed a decline in spirituality, attributing it to the overwhelming stress of their wives’ illness. They questioned their faith, feeling abandoned by God amidst their struggles, leading to a cessation of prayer and reduced church attendance. A study in Kenya^[25]^ similarly identified various coping strategies, including prayer, stoicism, and denial, among individuals grappling with the challenges of cervical cancer. Despite these spiritual fluctuations, family members eventually resumed their normal routines following the initial shock of the diagnosis.

Several men in the study noted a loss of interest in once-beloved recreational activities like outings and games, indicating a potential sign of depression. This shift in behavior underscores the importance of seeking medical and psychological support to address possible complications. These findings align with research on anhedonia,^[26]^ which highlights how a lack of enjoyment can hinder social connections and lead to withdrawal from social events. Declining invitations and avoiding gatherings may occur when individuals no longer derive happiness from such interactions, making social engagement challenging.

Many men expressed concern about their own health, particularly the risk of contracting an infection from their partners, especially with chronic conditions like cervical cancer. The majority of participants in this study feared acquiring cervical cancer through sexual intercourse. Providing education on cervical cancer causes and management, dispelling cancer myths, is crucial for addressing these fears. This study corresponds with research on the fear of cancer (carcinophobia),^[27]^ which found that individuals with personal experiences of cancer or knowing someone with cancer are more likely to fear contracting the disease.

This study revealed the significant role of social support networks. While some participants received financial assistance from relatives and friends, others did not receive any form of support but expressed readiness to accept it. Given the expensive and demanding nature of cervical cancer treatment, both financial and emotional support from family and friends are invaluable for partners of patients. This finding aligns with research in Kenya,^[25]^ which identified religion, family support, positive interpretations, and palliative care as coping mechanisms among cervical cancer patients and their caregivers.

Some participants effectively coped by employing diversional therapies for their partners. These included playing their partners’ favorite games, watching television (TV) programs together, and providing compact disks of beloved telenovelas for them to enjoy alone. These activities aimed to keep their partners occupied, uplifted, and enhance their sense of well-being. This aligns with a study^[28,29]^ involving cancer patients, where similar diversional therapies like watching TV and spending quality time together were found to aid coping.

Most Christians in the study exhibited persistent faith, relying on prayer to cope with their partners’ illnesses, encouraging both themselves and their partners to trust in God for healing. Similarly, Muslim participants displayed a similar attitude of faith in Allah, hoping for their partners’ recovery according to Allah’s will. This aligns with a study^[25]^ in Kenya, which identified prayer as a coping mechanism used by men to support their partners, consistent with the findings of this study. Likewise, research by^[23]^ on surviving cervical cancer among 15 participants found that praying and hoping for a miraculous healing from God served as coping strategies.

## 5. Conclusion

The result of the study suggests that the participants had fair knowledge on cervical cancer but most of them were facing financial crisis in the course of treatment since the treatment of this condition is expensive. However, almost every man adopted a strategy to cope with the condition.

## 6. Study limitation

Enlisting additional male participants for the study proved to be both taxing and challenging due to their limited availability within the study setting (hospital). Their commitments beyond visiting their wives at the hospital during the data collection period further complicated matters. The relatively substantial sample size, while beneficial, might influence the outcomes, thus potentially limiting the generalizability of this study. Nevertheless, exploring the transferability of the study could shed light on its applicability within a similar environment.

## Acknowledgments

On behalf of all the researchers, we wish to acknowledge all authors whose work was cited in this study, and the male partners who partook in this study.

## Author contributions

**Conceptualization:** Evans Appiah Osei, Elvis Frimpong, Samuel Kontoh.

**Data curation:** Elvis Frimpong, Hawa Amadu Toure, Manuela Akosua Menka.

**Formal analysis:** Evans Appiah Osei, Isabella Garti, Mary Ani-Amponsah, Jamilatu B. Kappiah, Samuel Kontoh.

**Investigation:** Evans Appiah Osei, Isabella Garti, Mary Ani-Amponsah, Jamilatu B. Kappiah, Manuela Akosua Menka, Samuel Kontoh.

**Methodology:** Evans Appiah Osei, Isabella Garti, Mary Ani-Amponsah, Elvis Frimpong, Hawa Amadu Toure, Jamilatu B. Kappiah, Manuela Akosua Menka, Samuel Kontoh.

**Software:** Evans Appiah Osei, Jamilatu B. Kappiah.

**Validation:** Evans Appiah Osei, Isabella Garti, Mary Ani-Amponsah, Jamilatu B. Kappiah.

**Visualization:** Evans Appiah Osei, Isabella Garti, Mary Ani-Amponsah, Hawa Amadu Toure, Jamilatu B. Kappiah.

**Writing – original draft:** Evans Appiah Osei, Isabella Garti, Mary Ani-Amponsah, Elvis Frimpong, Hawa Amadu Toure, Jamilatu B. Kappiah, Manuela Akosua Menka, Samuel Kontoh.

**Writing – review & editing:** Evans Appiah Osei, Isabella Garti, Mary Ani-Amponsah, Elvis Frimpong, Hawa Amadu Toure, Jamilatu B. Kappiah, Manuela Akosua Menka, Samuel Kontoh.
